# Sputum myeloperoxidase in chronic obstructive pulmonary disease

**DOI:** 10.1186/2047-783X-19-12

**Published:** 2014-03-03

**Authors:** Alling Zhu, Dehai Ge, Jingying Zhang, Yue Teng, Cheng Yuan, Mao Huang, Ian M Adcock, Peter J Barnes, Xin Yao

**Affiliations:** 1 Department of Respiratory Medicine, The First Affiliated Hospital of Nanjing Medical University, 300 Guangzhou Road, Nanjing 210029, China; 2 Department of Respiratory Medicine, Shanghai Meishan Hospital, Xinjian Street, Nanjing 210039, China; 3 Airway Disease Section, National Heart and Lung Institute, Imperial College, Dovehouse Street, London SW3 6LY, United Kingdom

**Keywords:** Chronic obstructive pulmonary disease, Myeloperoxidase, Sputum, Biomarker

## Abstract

**Background:**

Airway inflammation, especially neutrophilic airway inflammation, is a cardinal pathophysiologic feature in chronic obstructive pulmonary disease (COPD) patients. The ideal biomarkers characterizing the inflammation might have important potential clinical applications in disease assessment and therapeutic intervention. Sputum myeloperoxidase (MPO) is recognized as a marker of neutrophil activity. The purpose of this meta-analysis is to determine whether sputum MPO levels could reflect disease status or be regulated by regular medications for COPD.

**Methods:**

Studies were identified by searching PubMed, Embase, the Cochrane Database, CINAHL and http://www.controlled-trials.com for relevant reports published before September 2012. Observational studies comparing sputum MPO in COPD patients and healthy subjects or asthmatics, or within the COPD group, and studies comparing sputum MPO before and after treatment were all included. Data were independently extracted by two investigators and analyzed using STATA 10.0 software.

**Results:**

A total of 24 studies were included in the meta-analysis. Sputum MPO levels were increased in stable COPD patients when compared with normal controls, and this increase was especially pronounced during exacerbations as compared with MPO levels during the stable state. Theophylline treatment was able to reduce MPO levels in COPD patients, while glucocorticoid treatment failed to achieve the same result.

**Conclusion:**

Sputum MPO might be a promising biomarker for guiding COPD management; however, further investigations are needed to confirm this.

## Review

### Introduction

Chronic obstructive pulmonary disease (COPD) is a condition of chronic airflow limitation that is not completely reversible and is often progressive [1]. It is one of the leading causes of morbidity and mortality in the world, resulting in a growing social and economic burden [1].

Airway inflammation is a cardinal pathophysiologic feature in patients with COPD [2], and assessment of airway inflammation may therefore play an important role in the management of COPD and assessment of the disease status and prediction of prognosis. Additionally, oxidative damage in the lower respiratory tract accelerates the development of COPD, and oxidative stress levels are elevated in COPD patients [3].

Neutrophils are a major cellular component of COPD inflammation [4]. The number of neutrophils is increased in induced sputum and bronchoalveolar lavage fluid (BALF) of COPD patients, and correlates with disease severity [1,5]. Usually, the markers such as myeloperoxidase (MPO), interleukin-8 (IL-8), leukotriene B4 (LTB4), and human neutrophil lipocalin (HNL) [6] are used as the mediators of neutrophil activity. According to the Food and Drug Administration (FDA), ‘With the exception of lung function tests, there are no well validated biomarkers or surrogate endpoints that can be used to establish efficacy for a drug for COPD [7]. The marker MPO has been systematically studied in an attempt to determine if it is an effective biomarkers for COPD.

Myeloperoxidase (MPO) is a heme-containing peroxidase expressed abundantly in neutrophils and to a lesser extent in monocytes [8]. MPO is one the principal enzymes released from secondary granules following neutrophil activation [9,10]. Although the generation of oxidants by MPO is beneficial in terms of the immune response to invading pathogens, there is considerable evidence that inappropriate stimulation of oxidant formation can result in host tissue damage. Sputum derived from the surface of the lower respiratory tract mucus is a direct sample from the lower airways, and analysis of sputum composition makes non-invasive measurement of airway inflammation possible. Therefore, sputum MPO may be a potential non-invasive biomarker that reflects the severity or prognosis of COPD.

Conventional pharmacotherapy includes bronchodilator medications (β2 agonists, anticholinergics, and methylxanthines) and inhaled glucocorticosteroids (ICS). These can improve health status, and reduce symptoms and exacerbations [1]. However, previous treatment has not been shown statistically to decrease mortality [11,12]. Novel anti-inflammatory therapies are therefore being developed as potential therapeutic agents, a recent study [13] showed that an MPO inhibitor is able to stop progression of COPD in an animal model by inhibiting oxidative damage. The inhibition of MPO may be a novel and useful therapeutic treatment for COPD [13].

This is the first meta-analysis about sputum MPO in COPD patients. There have been many recent studies reporting changes in MPO level in COPD patients [14-19]; some of these studies failed to replicate the earlier results [15,16]. And there were few studies that contained large samples. Other studies [20-26] also observed the changes of MPO level before and after anti-inflammatory treatment, but results were inconsistent. We therefore performed the first meta-analysis of published studies examining sputum MPO in COPD patients to determine: 1) whether the MPO level is increased in COPD patients compared to healthy subjects or asthmatics; 2) whether there is a difference in MPO level between patients with stable disease and during acute exacerbation; and 3) whether anti-inflammatory treatment could reduce the MPO level.

## Materials and methods

Studies were identified in literature searches of PubMed (1966 to September 2012), Embase (1980 to September 2012), the Cochrane Database (1972 to September 2012), CINAHL (1981 to September 2012), and http://www.controlled-trials.com for relevant reports. We used the following keywords: (sputum OR mucus OR phlegm) AND (myeloperoxidase OR MPO) AND (Chronic Obstructive Pulmonary Disease OR obstructive OR bronchitis OR pulmonary emphysema OR bronchial hyper-reactivity OR COPD OR COLD OR emphysema OR airway obstruction OR airway inflammation), without language restrictions. Additionally, a manual search was conducted using all reviews on this topic, which provided bibliographies of original reports. Inclusion criteria were: 1) observational studies or randomized controlled trials (RCT); 2) studies comparing MPO level in COPD patients with healthy subjects or asthmatics or themselves; and 3) participants were adults with COPD as diagnosed by GOLD [1] or the American Thoracic Society or European Respiratory Society (ATS/ERS) standards [27].

Exclusion criteria were: 1) the MPO value in disease was provided without control values [28-33]; 2) the study did not produce original data [34-42]; or 3) the study investigated sputum MPO but not in COPD patients [43-45].

Two reviewers (AZ and DG) independently scanned the titles and abstract sections of all the articles retrieved based on the explicit criteria. Full articles were searched when the information met the inclusion criteria. Disagreements in extracted data were resolved by discussion with a third review author (XY).

Quality evaluation was conducted for each randomized controlled trial (RCT) with the Jadad criteria [46]. The trials with a score of 3 to 5 were enrolled in our study. For each accepted study, we extracted the following data, when available: the number of participants (*n*), age, gender, clinical features, treatment (s), method of sputum sampling, method of MPO measurement, MPO value. The MPO value was presented as mean value or standard deviation (SD), standard error of the mean (SEM), 95% confidence interval (95% CI), median or interquartile range and the percent predicted values of FEV_1_ (% pred FEV_1_) in each group. The SEM or 95% CI was transformed into SD, using a statistical formula. If the study provided medians and interquartile ranges, we imputed the means and SDs as described by Hozo *et al. *[47]. We calculated the lower and upper ends of the range by multiplying the difference between the median and upper and lower ends of the interquartile range by 2 and adding or subtracting the product from the median, respectively. If the article provided an MPO value in chart form, we used the Engauge Digitizer 4.1 software to estimate its value, and then calculated the mean and SD with SPSS software(Version 16.0, SPSS Corporation, College Station, Chicago, USA). For articles where data were not complete, the authors were contacted. Subject characteristics are given in more detail in Additional file 1 [see Additional file 1: Table S1-S3]. Analyses were performed using STATA software (Version 10.0, STATA Corporation, College Station, TX, USA). The standard mean difference (SMD) was chosen to combine statistics because the difference in mean value was too large and resulted in large heterogeneity. Summary estimates and the weightings for each outcome were evaluated based on the DerSimonian- Laird random effects model. Heterogeneity of study results was tested using the Mantel- Haenszel method, and results were considered heterogeneous if the *P* value was <0.05. We used the *I*^2^ statistic to measure the level of statistical heterogeneity for each outcome [48]. We used a fixed effects model when *I*^2^ was equal or less than 50%, and a random effects model when *I*^2^ was greater than 50%. Publication bias was evaluated through visual inspection of funnel plots, using the Begg’s test [49] and the Egger’s Asymmetry test [50]. Publication bias was assumed to be present if the *P* value was less than 0.05.

## Results

**Figure 1:**
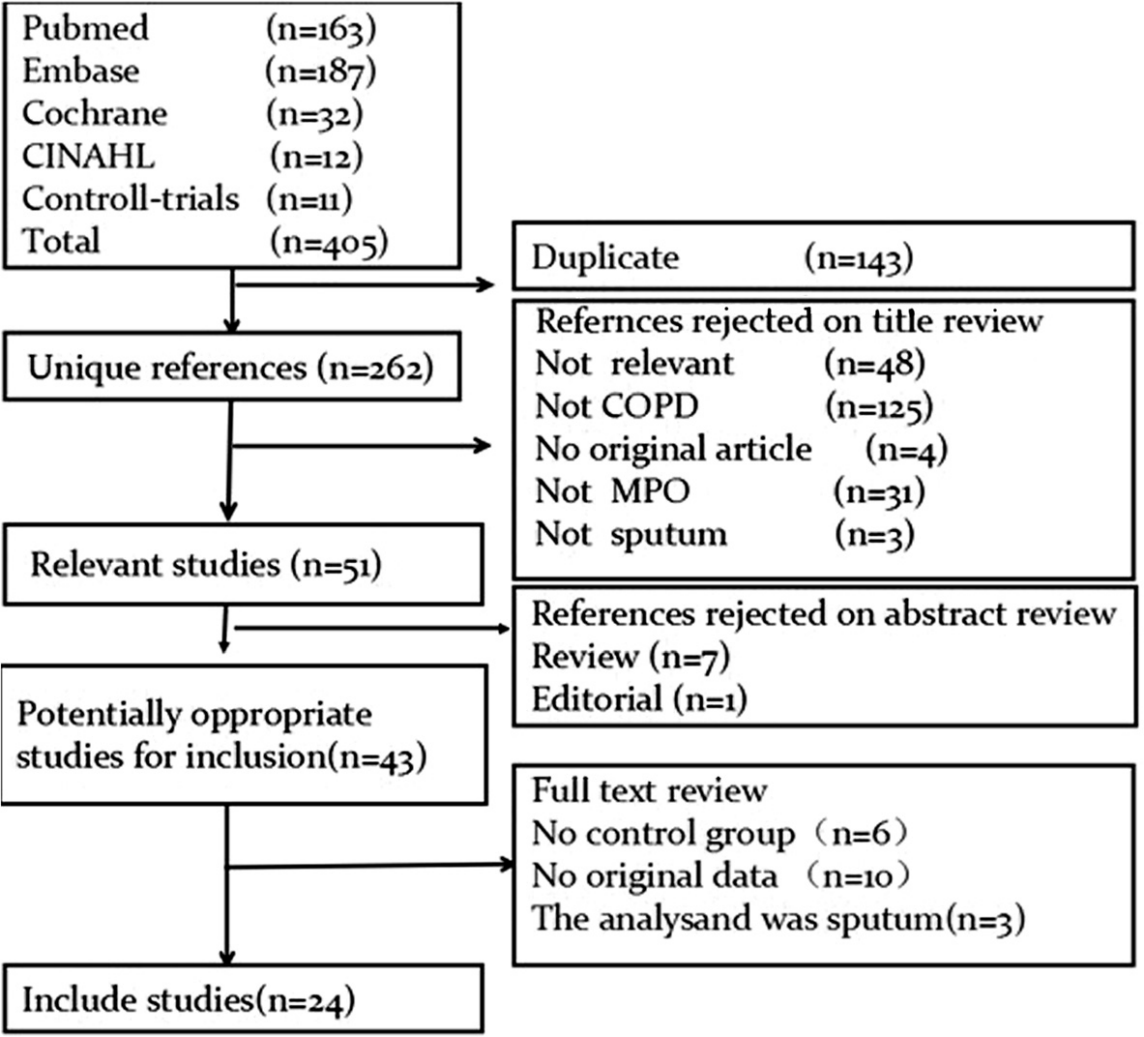
Results of the systematic literature search.

In the primary literature search, 405 records were identified, of which 262 were unique. After screening the titles and abstracts, 219 studies were excluded because they were either animal studies, review articles, or irrelevant to the current analysis, yielding 43 candidate studies. Of the 43 reports selected for detailed evaluation, 19 studies were further excluded for various reasons (Figure 1). Finally, 24 articles were included in our meta-analysis.

**Figure 2:**
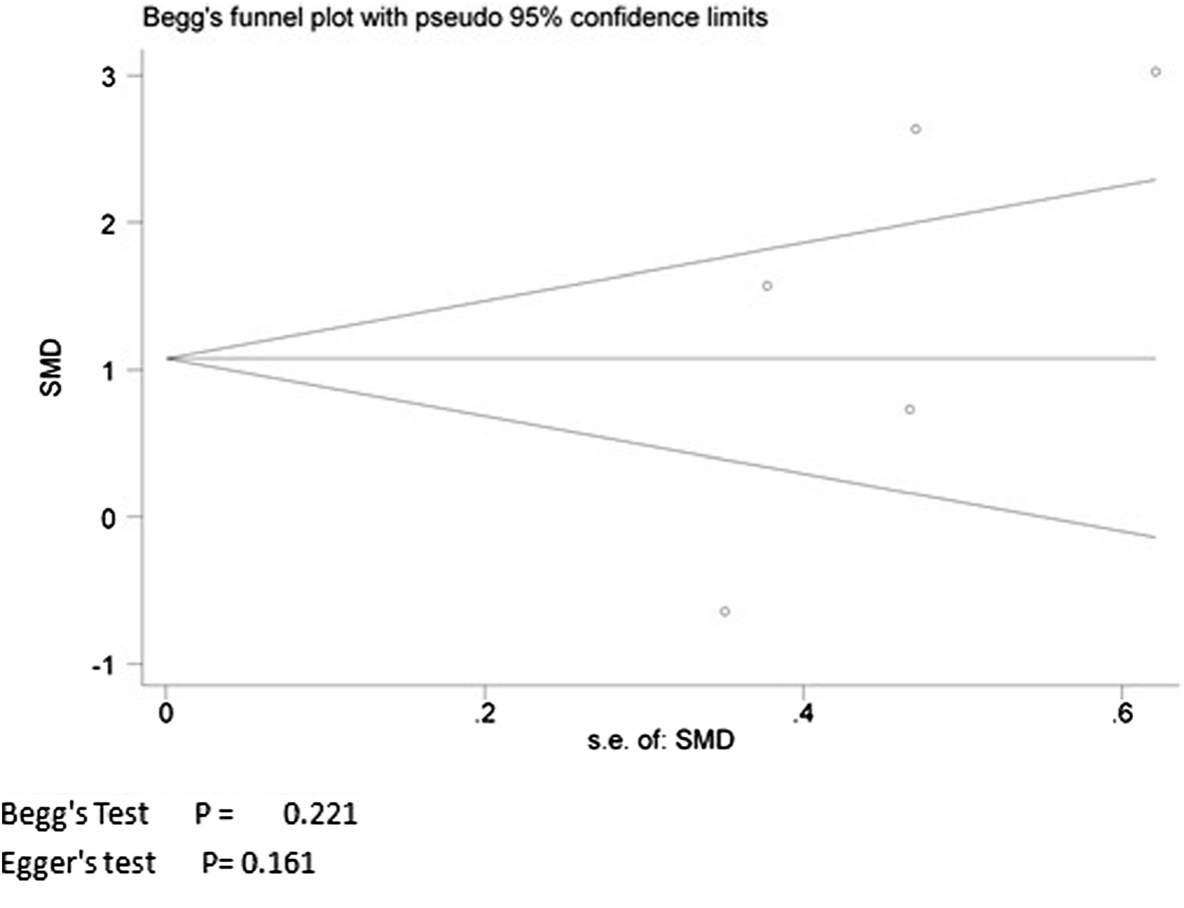
Publication Bias.

Among the 24 studies, three directly provided SD, nine studies used the SEM. We transformed the SEM from these nine studies into SD. Four studies used medians and the other four studies provided interquartile ranges, with the one remaining study providing 95% CI. We inputted these into SDs. In addition, we used the Engauge Digitizer 4.1 software to estimate the MPO value, and then used SPSS software to calculate the mean value of the MPO level in the other three studies (data are presented as mean ± SD). All studies provided basic information and detailed methodology for patients and controls. The characteristics of these studies were listed in Additional file 1. There was no evidence of publication bias (Begg’s Test: *P* = 0.221; Egger’s test: *P* = 0.161). The Funnel plot is shown in Figure 2.

1. MPO levels in COPD patients and healthy subjects.

**Figure 3:**
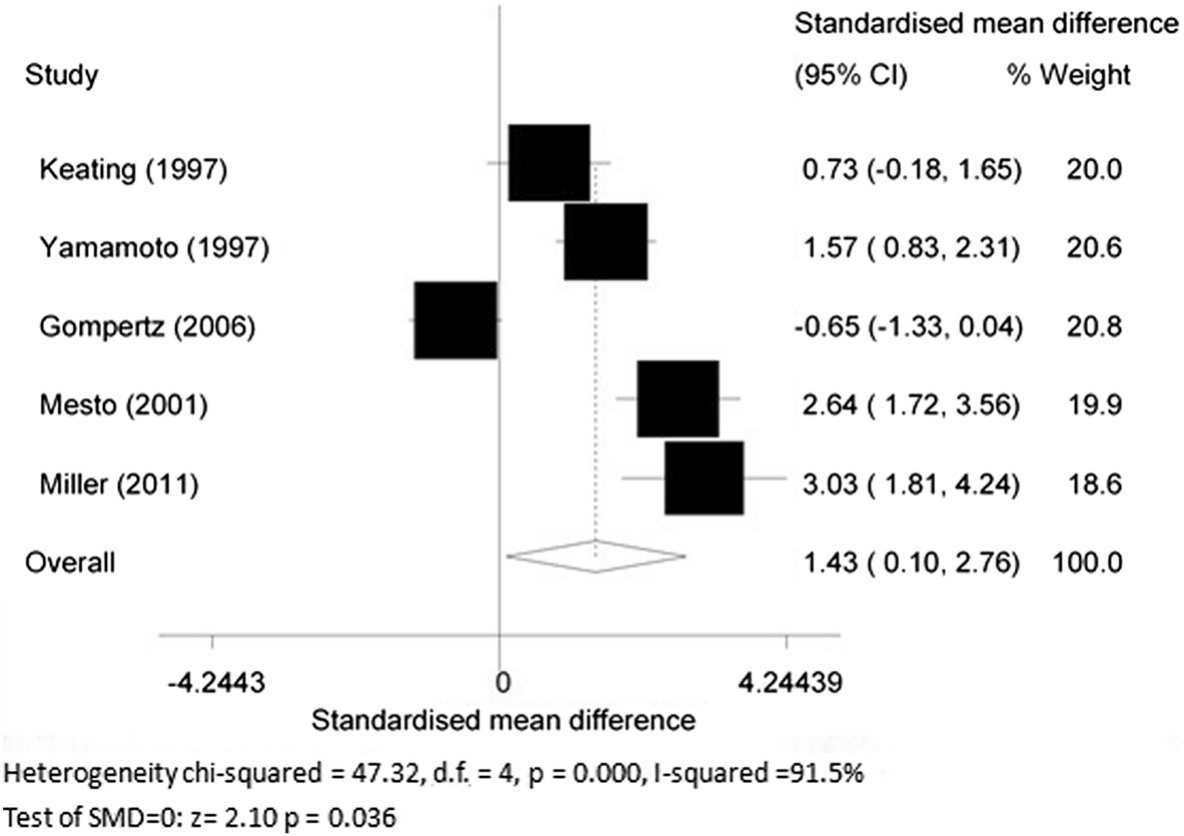
Myeloperoxidase (MPO) levels in chronic obstructive pulmonary disease (COPD) patients and healthy subjects.

Data were extracted from five articles [14-16,51,52] focusing on MPO levels in COPD patients compared to healthy subjects. The overall SMD of MPO levels was 1.43 (95% CI 0.10 to 2.76, *P* = 0.036; *I*^2^ = 91.5%; random effects model). COPD patients showed higher sputum MPO levels as compared to healthy subjects (Figure 3).

2. MPO levels in COPD patients and asthmatics.

**Figure 4:**
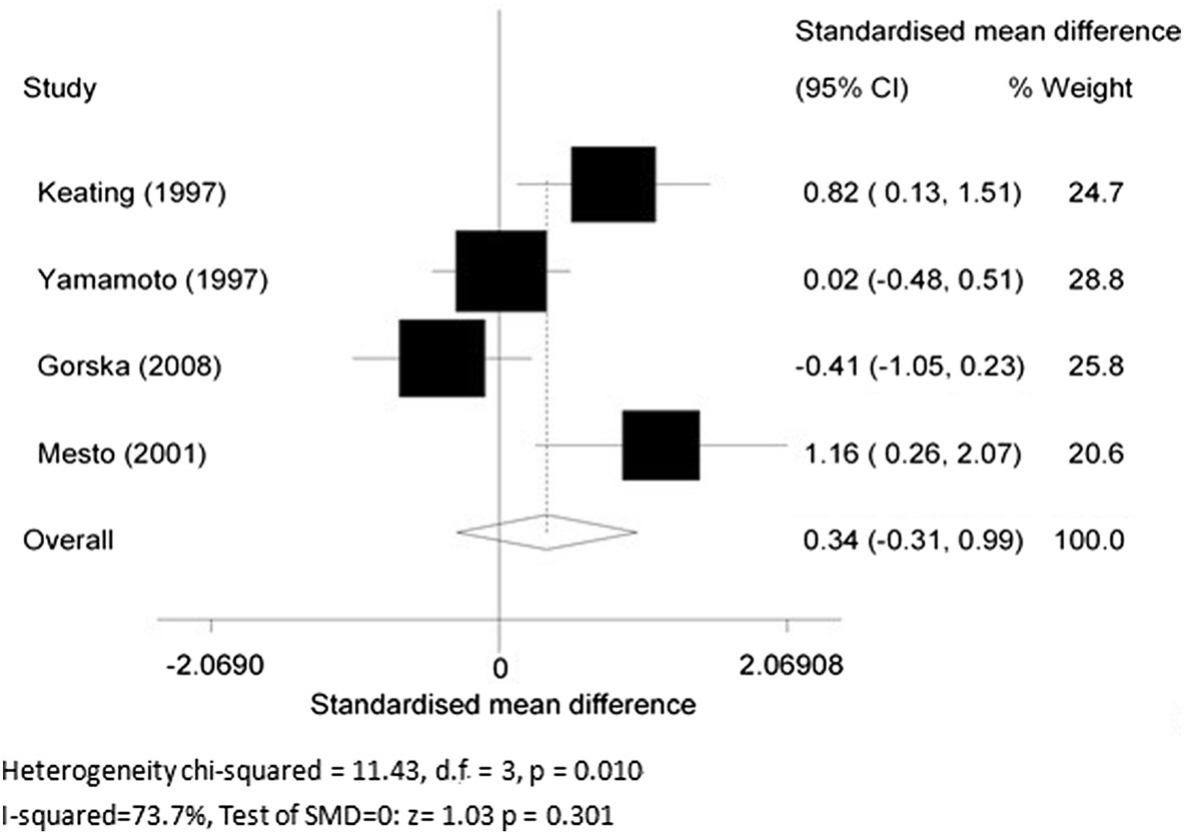
Myeloperoxidase (MPO) levels in chronic obstructive pulmonary disease (COPD) patients and asthmatics.

Four articles [14,15,17,51] compared MPO levels in COPD patients with asthmatics. The SMD of MPO levels between the two groups was 0.34 (95% CI -0.31 to 0.99, *P* = 0.301; *I*^2^ = 73.7%; random effects model). There was no statistically significant difference between COPD patients and asthmatics (Figure 4).

3. MPO concentrations in stable COPD and during acute exacerbations.

**Figure 5:**
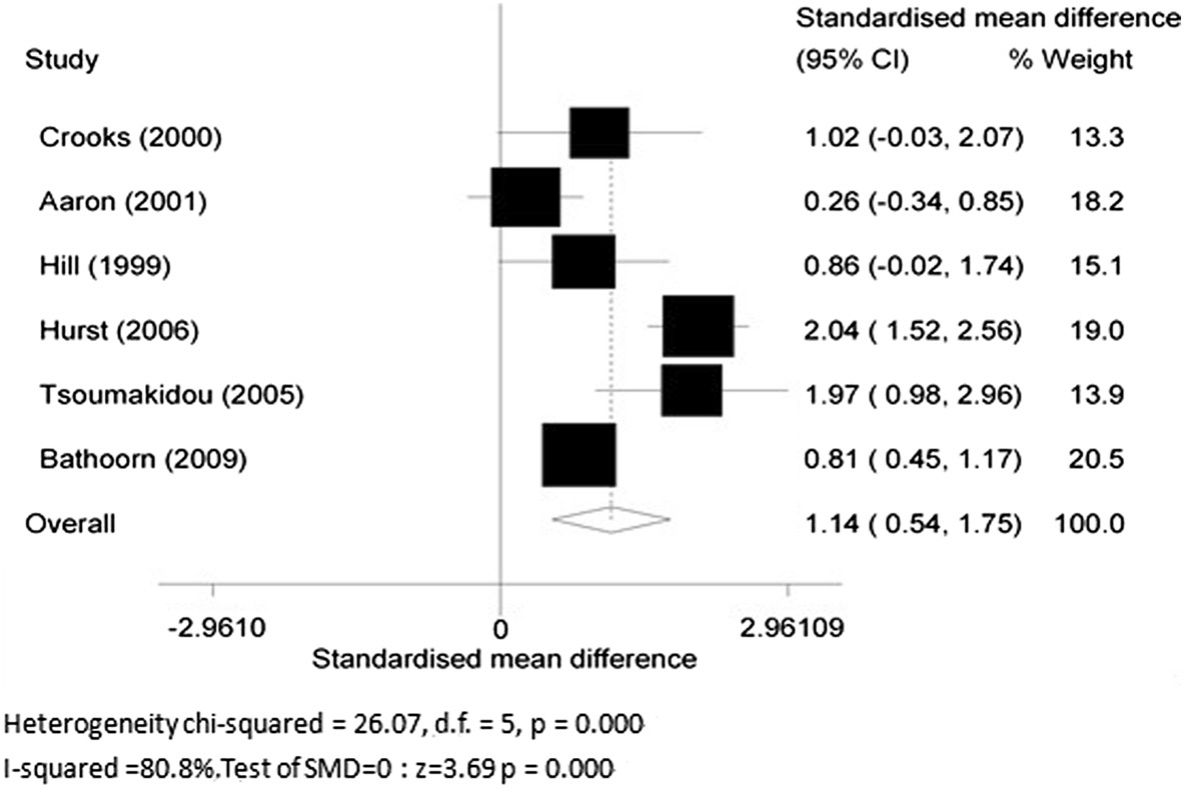
Myeloperoxidase (MPO) concentrations in stable chronic obstructive pulmonary disease and during acute exacerbation.

Six articles [18,19,53-56] focused on the difference in MPO levels in stable COPD patients and patients with exacerbations. Five of them compared the MPO levels within COPD patients before and after exacerbation, while the remaining study compared MPO levels in COPD patients with those in healthy controls. Therefore, the former five articles were chosen for subgroup analysis. The overall SMD of MPO levels was 1.06 (95% CI 0.17 to 1.95, *P* = 0.019; *I*^2^ = 90.6%; random effects model) between the stable COPD group and exacerbation group. COPD patients with exacerbation showed a significantly higher MPO level than stable COPD patients (Figure 5).

4. The changes of MPO level before and after theophylline treatment.

**Figure 6:**
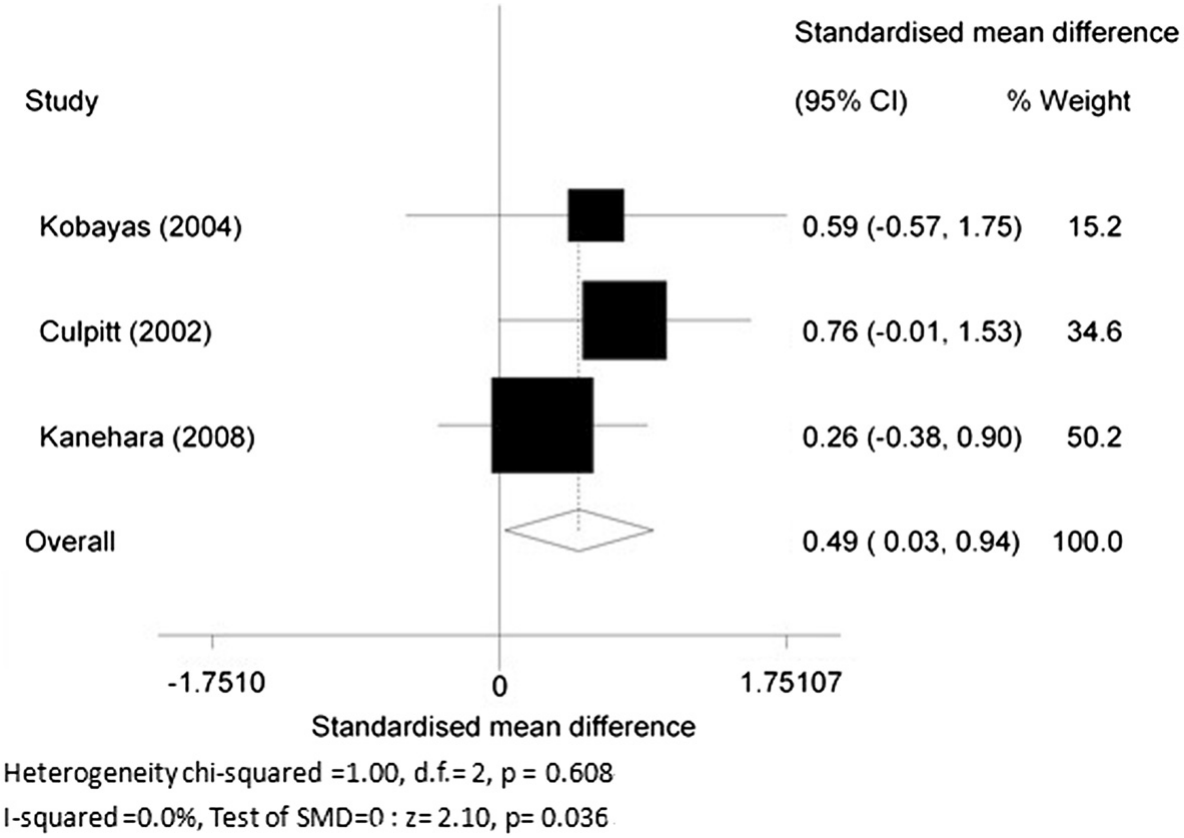
The changes before and after theophylline treatment.

Three RCT articles [20-22] compared changes of MPO levels before and after theophylline treatment. The SMD was 0.49 (95% CI 0.03 to 0.94, *P* = 0.036; *I*^2^ = 0.0%; fixed effects model), indicating theophylline treatment could reduce MPO levels in sputum of COPD patients (Figure 6).

5. The changes of MPO level before and after glucocorticoid treatment.

**Figure 7:**
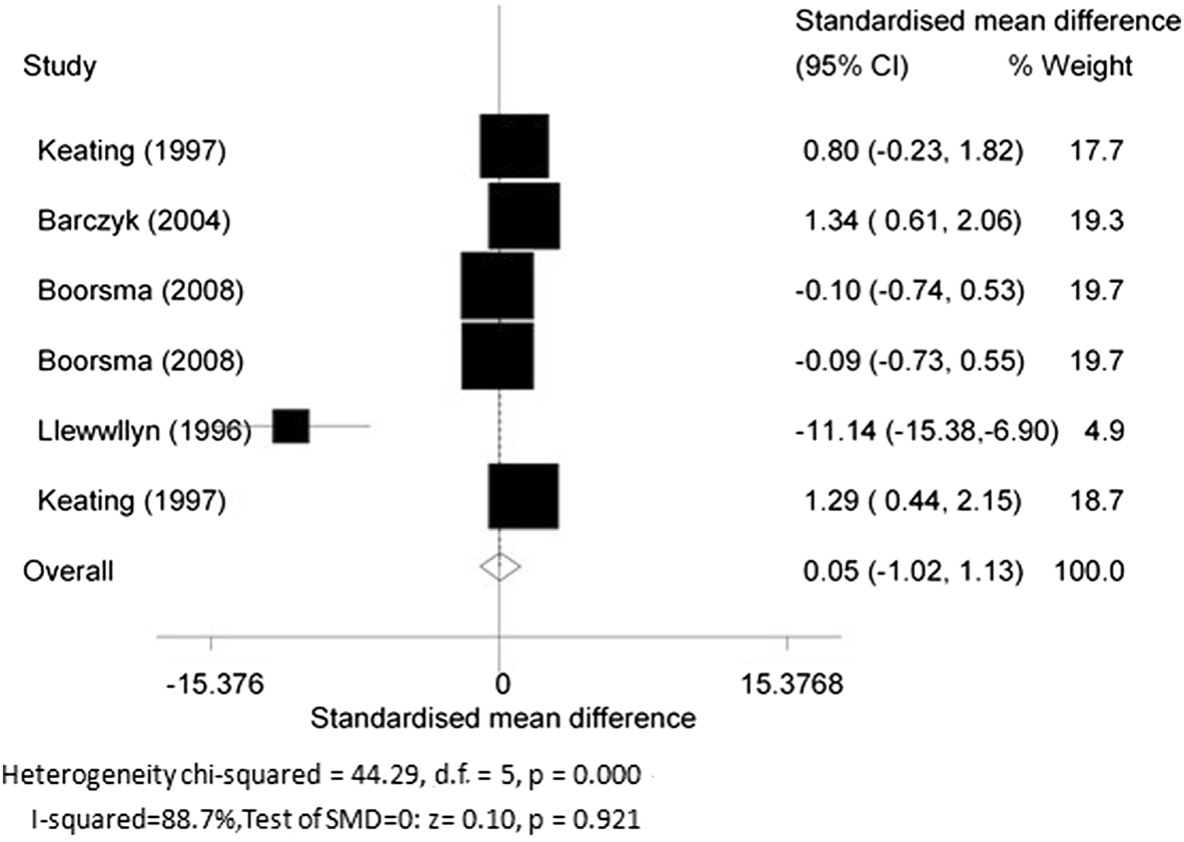
The changes of myeloperoxidase (MPO) before and after glucocorticoid treatment.

Four RCT articles [23-26] studied the effect of steroid treatment (including inhaled and oral corticosteroids) on the sputum MPO value. The SMD of MPO in COPD patients before and after glucocorticoid treatment was 0.05 (95% CI-1.02 to 1.13, *P* = 0.921; *I*^2^ = 88.7%; random effects model) indicating that glucocorticoids had no effect on MPO levels in sputum of COPD patients (Figure 7).

6. The changes of MPO level before and after other treatments.

Gompertz *et al*. [57] and Gronke *et al.*[58] showed a small decrease in sputum MPO level in the patients treated with LTB4 receptor antagonists on sputum MPO levels in COPD patients. And the difference had no statistical significance (*P* = 0.06). The other three studies [44,59,60] also showed no difference in patients treated with macrolide antibiotics and tulobuterol.

7. The changes of MPO levels between COPD patients with and without homozygous (PiZ) α1-antitrypsin (AAT) deficiency.

**Figure 8:**
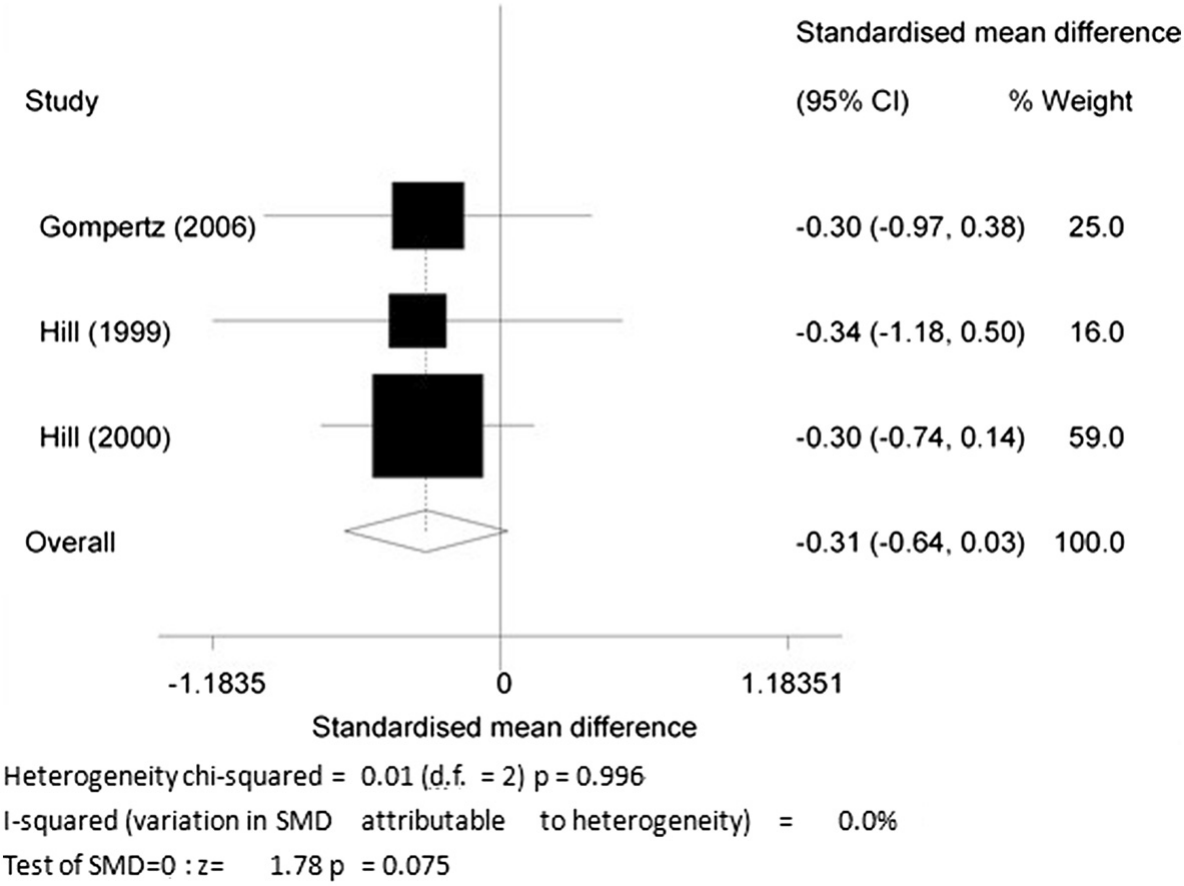
The changes of myeloperoxidase (MPO) levels in chronic obstructive pulmonary disease (COPD) patients with and without homozygous (PiZ) α1-antitrypsin (AAT) deficiency.

Data were acquired from three articles [16,53,61], where COPD patients without homozygous (PiZ) α1-antitrypsin (AAT) deficiency were compared with COPD patients with AAT deficiency. The overall SMD was -0.31 (95% CI-0.64 to 0.03, P = 0.075; I^2^ = 0%; fixed effects model) (Figure 8), indicating that there was no significant difference between the two groups.

## Discussion

This meta-analysis demonstrated that sputum MPO levels were significantly increased in stable COPD patients as compared with normal controls but not different from those seen in asthmatic subjects (*P* = 0.301). It also indicated that MPO levels were also markedly increased during exacerbations compared to the stable state. Theophylline treatment significantly reduced sputum MPO levels, while glucocorticoid treatment had no effect. In addition, the COPD subjects with AAT deficiency appeared to have higher sputum MPO levels than those with normal AAT, although this did not reach statistical significance.

The elevation of sputum MPO levels in COPD patients correlated with the increased number of neutrophils in their respiratory tract [62], indicating that neutrophil activation was associated with degranulation of the primary and secondary granules. MPO is considered to contribute substantially to the microbicidal activity of neutrophils and monocytes through the generation of reactive oxidant species [10]. In particular, the combination of MPO, its substrate hydrogen peroxide (H_2_O_2_), and a halide results in a powerful and effective antimicrobial system [63]. MPO has also been implicated in the induction of lung injury. For example, while the infusion of glucose oxidase or peroxidase alone produced little damage in *in vivo* studies, the combination of the two resulted in interstitial fibrosis [64]. MPO release results in increased amounts of H_2_O_2_ induction, which is compatible with the MPO system playing a role in epithelial cell injury in man [65]. As such, MPO has opposing roles as an effective antimicrobial which can, to some degree, be replaced and as a detrimental agent that has the potential to produce damage and contribute to disease [9]. Perhaps, interventions that reduce MPO level might be anticipated to modify the progression of COPD.

It is known that inflammation seen in asthma is different from that seen in COPD with inflammation from asthma being predominantly eosinophilic rather than neutrophilic bronchitis [66]. As such, the levels of sputum MPO in COPD patients was higher than that in asthma patients. However, the difference was not significant, and the results may suggest that activation of neutrophils may also occur in asthmatics [14] or that the presence of chronic expectoration in COPD patients may complicate sputum analysis. Of course, more research and further work in this area is needed.

Our study has shown that COPD patients with exacerbations had elevated sputum MPO levels relative to their clinically stable state. This is consistent with the fact that COPD exacerbations, especially in bacterial exacerbations [19], are triggered by neutrophilic inflammation [40]. Apart from the study of Fens *et al. *[67], which suggested that there was no significant difference in sputum MPO level between GOLD stage I and II, few studies have investigated whether MPO levels are associated with disease severity. Further studies are required to determine this.

Previous studies showed that theophylline induces neutrophil apoptosis through adenosine A_2A_ receptor antagonism [68] and by reduced O_2_^-^ production in both granulocytes and eosinophils [69]. Liboshi *et al. *[70] suggested that long-term treatment with theophylline reduced inflammatory cytokines and neutrophils in the sputum of COPD patients. Theophylline, therefore, has an inhibitory action on airway inflammation, particularly in relation to the activation of inflammatory cells including neutrophils. This may explain why theophylline significantly reduced the sputum levels of MPO in COPD patients.

Interestingly, glucocorticoid treatment was found to have no effect on sputum MPO level in COPD patients, regardless of the route of administration and dosage, which further demonstrated that airway neutrophilic inflammation is not responsive to glucocorticoids [25]. Although clinically, some COPD patients could benefit from glucocorticoids, it might be due to the inhibition of the ‘eosinophilic component’ of the inflammatory process in the airways by glucocorticoids [25]. This may explain why COPD patients with neutrophil inflammation do not respond well to steroids. This may also explain the discrepancies between different studies regarding glucocorticoid effects in the treatment of airway anti-inflammation in COPD patients [71,72]. More recent laboratory studies suggest that low-dose theophylline may reverse steroid resistance by enhancing decreased histone deacetylase (HDAC)-2 activity [73]. However, no study confirmed whether combined medications could affect the MPO level in COPD patients. More translational research in this area is clearly required.

Two studies [57,58] determined the effect of oral leukotriene B_4_ (LTB_4_) receptor antagonists on sputum MPO levels in COPD patients. Gompertz *et al. *[57] showed a decrease in sputum MPO level in the patients treated with BAYx1005, perhaps because LTB4 can induce the migration of neutrophils into the lungs [74] and play a role in leukocyte degranulation [75]. But this failed to achieve statistical significance. Furthermore, the reproducibility of repeated measurements of MPO in the Gronke *et al. *[58] study was also less than satisfactory. These data failed to demonstrate that sputum MPO levels, and by implication, airway inflammation in COPD are modulated by LTB4 receptor antagonists.

Two additional studies [59,60] evaluated the anti-inflammatory effect of macrolide antibiotics on sputum MPO levels in COPD patients. The course of treatment varied from 10 days to 12 months. There was no significant difference in sputum MPO levels regardless of the duration of treatment. In addition, Kanehara *et al. *[22] also demonstrated no significant effect of tulobuterol on sputum MPO levels in mild-to-moderate COPD. Overall, these data suggest that only theophylline has a consistent effect on sputum MPO levels. Larger studies will need to be performed to determine whether other anti-inflammatory agents affect sputum MPO.

COPD subjects with AAT deficiency appear to have higher sputum MPO levels, probably because these patients may associate with a general increase in the concentrations of several neutrophilic airway inflammatory parameters. Hubbard *et al.* suggested that alveolar macrophage release of LTB_4_, the major chemoattractant responsible for the increased neutrophil migration, is increased in AAT deficiency as a result of free elastase activity [76]. The latter can reduce the secretion of secretory leukoprotease inhibitor (SLPI) [77], as well as the increase in the permeability of airway cells. However, recent studies have indicated that the relationship between LTB_4_ and MPO is less clear [41]. No statistically significant difference in the sputum MPO level was observed between COPD patients with AAT deficiency and those with normal AAT levels in our study. However, more studies are needed to confirm this.

Our meta-analysis has some limitations. The most obvious one is that the methods of collecting and treating sputum varied in different studies, and the few studies available prevented subgroup analyses. Another major limitation is the lack of standardization of sputum MPO measurement. The MPO level or its activity was assessed by radioimmunoassay (RIA) or enzyme-linked immunosorbent assay (ELISA) or the substrate O-dianisidine dihydrochloride assay, so the difference in mean values was large, resulting in a large heterogeneity, and we failed to perform the metaregression because of few studies. Ideally, further large-sample studies are needed to address the role of MPO in COPD patients.

## Conclusions

Our study showed a higher MPO level in the sputum of COPD patients compared with control subjects, especially during exacerbations. Theophylline decreased sputum inflammation, especially neutrophilic inflammation, which was accompanied by a decrease in sputum MPO level. Further investigations are needed to determine whether sputum MPO levels alter according to disease severity. MPO might be a potentially useful non-invasive biomarker of estimating airway inflammation in guiding COPD patients’ treatment. The sputum MPO level will contribute to assess the occurrence of any exacerbations. The inhibition of MPO may be a promising therapeutic treatment for COPD.

## Abbreviations

AAT: α1-antitrypsindeficiency; BALF: bronchoalveolar lavage fluid; COPD: chronic obstructive pulmonary disease; ELISA: enzyme-linked immunosorbent assay; ICS: inhaled glucocorticosteroids LTB_4_, leukotriene B_4_; MPO: myeloperoxidase; RCT: randomize controlled trial; RIA: radioimmunoassay; SD: standard deviation; SEM: standard error of mean; SMD: standard mean difference; WMD: weighted mean difference; 95% CI: 95% confidence interval.

## Competing interests

The authors declare that they have no competing interests.

## Authors’ contributions

All authors read and met ICMJE criteria for authorship: AZ, DG, XY and MH participated in the design of this study; AZ, XY and JZ extracted data; AZ, XY, performed the analysis; AZ wrote the first draft of the manuscript and XY, IMA and PJB critically revised the manuscript. All authors read and approved the final manuscript.

## Supplementary Material

Additional file 1 **Table S1.** Studies included in the meta-analysis examining myeloperoxidase (MPO) levels in chronic obstructive pulmonary disease (COPD) patients and healthy subjects and asthmatics. Data are expressed as *mean ± SEM; #mean ± SD; &medians; ^ interquartile ranges; $:95% confidence interval. M, male; F, female; n, the number of participants; FEV1, forced expiratory volume in one second; ELISA, enzyme-linked immunosorbent assay; DDT, dithiothreitol. **Table S2.** Studies included in the meta-analysis examining myeloperoxidase (MPO) levels in stable COPD and during acute exacerbations. Data are expressed as *mean ± SEM; #mean ± SD; &medians; ^ interquartile ranges; $:95% confidence interval. COPD, chronic obstructive pulmonary disease; M, male; F, female; n, the number of participants; FEV1, forced expiratory volume in one second; AAT, α1-antitrypsindeficiency; ELISA, enzyme-linked immunosorbent assay; ICS, inhaled corticosteroids. **Table S3.** Studies included in the meta-analysis examining myeloperoxidase (MPO) levels in COPD patients before and after medicine treatment. Data are expressed as *mean ± SEM; #mean ± SD; &medians; ^ interquartile ranges; $:95% confidence interval. COPD, chronic obstructive pulmonary disease; M, male; F, female; n, the number of participants; FEV1, forced expiratory volume in one second; ELISA, enzyme-linked immunosorbent assay. Click here for file

## References

[B1] Rabe KF Hurd S Anzueto A Barnes PJ Buist SA Calverley P Fukuchi Y Jenkins C Rodriguez-Roisin R van Weel C Zielinski J Global Initiative for Chronic Obstructive Lung Disease Global strategy for the diagnosis, management, and prevention of chronic obstructive pulmonary disease: GOLD executive summary Am J Respir Crit Care Med 2007 176 532 555 17507545 10.1164/rccm.200703-456SO

[B2] Gross NJ Airway inflammation in COPD. Reality or myth? Chest 1995 107 210S 213S 7743829 10.1378/chest.107.5_supplement.210s

[B3] Zeng M Li Y Jiang Y Lu G Huang X Guan K Local and systemic oxidative stress status in chronic obstructive pulmonary disease patients Can Respir J 2013 20 35 41 23457673 10.1155/2013/985382 PMC3628645

[B4] Pettersen CA Adler KB Airways inflammation and COPD: epithelial-neutrophil interactions Chest 2002 121 5 Suppl 142S 150S 12010843 10.1378/chest.121.5_suppl.142s

[B5] Rovina N Dima E Gerassimou C Kollintza A Gratziou C Roussos C Interleukin-18 in induced sputum: association with lung function in chronic obstructive pulmonary disease Respir Med 2009 103 1056 1062 19208460 10.1016/j.rmed.2009.01.011

[B6] Xu SY Carlson M Engstrom A Garcia R Peterson CG Venge P Purification and characterization of a human neutrophil lipocalin (HNL) from the secondary granules of human neutrophils Scand J Clin Lab Invest 1994 54 365 376 7997842 10.3109/00365519409088436

[B7] U.S. Department of Health and Human Services Food and Drug Administration Center for Drug Evaluation and Research (CDER) Guidance for industry chronic obstructive pulmonary disease: developing drugs for treatment [http://www.fda.gov/downloads/drugs/guidancecomplianceregulatoryinformation/guidances/ucm071575.pdf]

[B8] van der Veen BS de Winther MP Heeringa P Myeloperoxidase: molecular mechanisms of action and their relevance to human health and disease Antioxid Redox Signal 2009 11 2899 2937 19622015 10.1089/ars.2009.2538

[B9] Klebanoff SJ Myeloperoxidase: friend and foe J Leukoc Biol 2005 77 598 625 15689384 10.1189/jlb.1204697

[B10] Hampton MB Kettle AJ Winterbourn CC Inside the neutrophil phagosome: oxidants, myeloperoxidase, and bacterial killing Blood 1998 92 3007 3017 9787133

[B11] Calverley PM Anderson JA Celli B Ferguson GT Jenkins C Jones PW Yates JC Vestbo J Salmeterol and fluticasone propionate and survival in chronic obstructive pulmonary disease N Engl J Med 2007 356 775 789 17314337 10.1056/NEJMoa063070

[B12] Tashkin DP Celli B Senn S Burkhart D Kesten S Menjoge S Decramer M A 4-year trial of tiotropium in chronic obstructive pulmonary disease N Engl J Med 2008 359 1543 1554 18836213 10.1056/NEJMoa0805800

[B13] Churg A Marshall CV Sin DD Bolton S Zhou S Thain K Cadogan EB Maltby J Soars MG Mallinder PR Wright JL Late intervention with a myeloperoxidase inhibitor stops progression of experimental chronic obstructive pulmonary disease Am J Respir Crit Care Med 2012 185 34 43 21997333 10.1164/rccm.201103-0468OC

[B14] Keatings VM Barnes PJ Granulocyte activation markers in induced sputum: comparison between chronic obstructive pulmonary disease, asthma, and normal subjects Am J Respir Crit Care Med 1997 155 449 453 9032177 10.1164/ajrccm.155.2.9032177

[B15] Yamamoto C Yoneda T Yoshikawa M Fu A Tokuyama T Tsukaguchi K Narita N Airway inflammation in COPD assessed by sputum levels of interleukin-8 Chest 1997 112 505 510 9266891 10.1378/chest.112.2.505

[B16] Gompertz S Hill AT Bayley DL Stockley RA Effect of expectoration on inflammation in induced sputum in alpha-1-antitrypsin deficiency Respir Med 2006 100 1094 1099 16257194 10.1016/j.rmed.2005.09.024

[B17] Gorska K Krenke R Domagala-Kulawik J Korczynski P Nejman-Gryz P Kosciuch J Hildebrand K Chazan R Comparison of cellular and biochemical markers of airway inflammation in patients with mild-to-moderate asthma and chronic obstructive pulmonary disease: an induced sputum and bronchoalveolar lavage fluid study J Physiol Pharmacol 2008 59 Suppl 6 271 283 19218651

[B18] Crooks SW Bayley DL Hill SL Stockley RA Bronchial inflammation in acute bacterial exacerbations of chronic bronchitis: the role of leukotriene B4 Eur Respir J 2000 15 274 280 10706491 10.1034/j.1399-3003.2000.15b09.x

[B19] Aaron SD Angel JB Lunau M Wright K Fex C Le Saux N Dales RE Granulocyte inflammatory markers and airway infection during acute exacerbation of chronic obstructive pulmonary disease Am J Respir Crit Care Med 2001 163 349 355 11179105 10.1164/ajrccm.163.2.2003122

[B20] Kobayashi M Nasuhara Y Betsuyaku T Shibuya E Tanino Y Tanino M Takamura K Nagai K Hosokawa T Nishimura M Effect of low-dose theophylline on airway inflammation in COPD Respirology 2004 9 249 254 15182277 10.1111/j.1440-1843.2004.00573.x

[B21] Culpitt SV de Matos C Russell RE Donnelly LE Rogers DF Barnes PJ Effect of theophylline on induced sputum inflammatory indices and neutrophil chemotaxis in chronic obstructive pulmonary disease Am J Resp Crit Care Med 2002 165 1371 1376 12016098 10.1164/rccm.2105106

[B22] Kanehara M Yokoyama A Tomoda Y Shiota N Iwamoto H Ishikawa N Taooka Y Haruta Y Hattori N Kohno N Anti-inflammatory effects and clinical efficacy of theophylline and tulobuterol in mild-to-moderate chronic obstructive pulmonary disease Pulm Pharmacol Ther 2008 21 874 878 18983928 10.1016/j.pupt.2008.09.003

[B23] Keatings VM Jatakanon A Worsdell YM Barnes PJ Effects of inhaled and oral glucocorticoids on inflammatory indices in asthma and COPD Am J Respir Crit Care Med 1997 155 542 548 9032192 10.1164/ajrccm.155.2.9032192

[B24] Barczyk A Sozanska E Trzaska M Pierzchala W Decreased levels of myeloperoxidase in induced sputum of patients with COPD after treatment with oral glucocorticoids Chest 2004 126 389 393 15302722 10.1378/chest.126.2.389

[B25] Boorsma M Lutter R Van De Pol MA Out TA Jansen HM Jonkers RE Long-term effects of budesonide on inflammatory status in COPD COPD 2008 5 97 104 18415808 10.1080/15412550801941000

[B26] Llewellyn-Jones CG Harris TAJ Stockley RA Effect of fluticasone propionate on sputum of patients with chronic bronchitis and emphysema Am J Respir Crit Care Med 1996 153 616 621 8564107 10.1164/ajrccm.153.2.8564107

[B27] Stoller JK Snider GL Brantly ML Fallat RJ Stockley RA Turino GM Konietzko N Dirksen A Eden E Luisetti M Stolk J Strange C American Thoracic Society; European Respiratory Society American Thoracic Society/European Respiratory Society Statement: standards for the diagnosis and management of individuals with alpha-1 antitrypsin deficiency Pneumologie 2005 59 36 68 15685488 10.1055/s-2004-830176

[B28] O’Donnell C Newbold P White P Thong B Stone H Stockley RA 3-Chlorotyrosine in sputum of COPD patients: relationship with airway inflammation COPD 2010 7 411 417 21166629 10.3109/15412555.2010.528086

[B29] Sapey E Bayley D Ahmad A Newbold P Snell N Stockley RA Inter-relationships between inflammatory markers in patients with stable COPD with bronchitis: intra-patient and inter-patient variability Thorax 2008 63 493 499 18057097 10.1136/thx.2007.086751

[B30] Bresser P Out TA van Alphen L Jansen HM Lutter R Airway inflammation in nonobstructive and obstructive chronic bronchitis with chronic haemophilus influenzae airway infection. Comparison with noninfected patients with chronic obstructive pulmonary disease Am J Respir Crit Care Med 2000 162 947 952 10988111 10.1164/ajrccm.162.3.9908103

[B31] Wilkinson TMA Donaldson GC Johnston SL Openshaw PJM Wedzicha JA Respiratory syncytial virus, airway inflammation, and FEV1 decline in patients with chronic obstructive pulmonary disease Am J Respir Crit Care Med 2006 173 871 876 16456141 10.1164/rccm.200509-1489OC

[B32] Gompertz S Bayley DL Hill SL Stockley RA Relationship between airway inflammation and the frequency of exacerbations in patients with smoking related COPD Thorax 2001 56 36 41 11120902 10.1136/thorax.56.1.36 PMC1745913

[B33] Stockley RA Bayley D Hill SL Hill AT Crooks S Campbell EJ Assessment of airway neutrophils by sputum colour: correlation with airways inflammation Thorax 2001 56 366 372 11312405 10.1136/thorax.56.5.366 PMC1746057

[B34] Schoonbrood DF Out TA Lutter R Reimert CM van Overveld FJ Jansen HM Plasma protein leakage and local secretion of proteins assessed in sputum in asthma and COPD. The effect of inhaled corticosteroids Clin Chim Acta 1995 240 163 178 8548926 10.1016/0009-8981(95)06139-0

[B35] Liesker JJ Bathoorn E Postma DS Vonk JM Timens W Kerstjens HA Sputum inflammation predicts exacerbations after cessation of inhaled corticosteroids in COPD Respir Med 2011 105 1853 1860 21802933 10.1016/j.rmed.2011.07.002

[B36] Rytila P Rehn T Ilumets H Rouhos A Sovijarvi A Myllarniemi M Kinnula VL Increased oxidative stress in asymptomatic current chronic smokers and GOLD stage 0 COPD Respir Res 2006 7 69 16646959 10.1186/1465-9921-7-69 PMC1524947

[B37] Powrie DJ Wilkinson TMA Donaldson GC Jones P Scrine K Viel K Kesten S Wedzicha JA Effect of tiotropium on sputum and serum inflammatory markers and exacerbations in COPD Eur Respir J 2007 30 472 478 17504798 10.1183/09031936.00023907

[B38] Parr DG White AJ Bayley DL Guest PJ Stockley RA Inflammation in sputum relates to progression of disease in subjects with COPD: a prospective descriptive study Respir Res 2006 7 136 17112387 10.1186/1465-9921-7-136 PMC1664562

[B39] Metso T Venge P Haahtela T Peterson CGB Seveus L Cell specific markers for eosinophils and neutrophils in sputum and bronchoalveolar lavage fluid of patients with respiratory conditions and healthy subjects Thorax 2002 57 449 451 11978925 10.1136/thorax.57.5.449 PMC1746329

[B40] Balbi B Bason C Balleari E Fiasella F Pesci A Ghio R Fabiano F Increased bronchoalveolar granulocytes and granulocyte/macrophage colony-stimulating factor during exacerbations of chronic bronchitis Eur Respir J 1997 10 846 850 9150323

[B41] Hill AT Bayley D Stockley RA The interrelationship of sputum inflammatory markers in patients with chronic bronchitis Am J Respir Crit Care Med 1999 160 893 898 10471615 10.1164/ajrccm.160.3.9901091

[B42] Hill AT Campbell EJ Hill SL Bayley DL Stockley RA Association between airway bacterial load and markers of airway inflammation in patients with stable chronic bronchitis Am J Med 2000 109 288 295 10996579 10.1016/s0002-9343(00)00507-6

[B43] Gompertz S O’Brien C Bayley DL Hill SL Stockley RA Changes in bronchial inflammation during acute exacerbations of chronic bronchitis Eur Respir J 2001 17 1112 1119 11491152 10.1183/09031936.01.99114901

[B44] White AJ Gompertz S Bayley DL Hill SL O’Brien C Unsal I Stockley RA Resolution of bronchial inflammation is related to bacterial eradication following treatment of exacerbations of chronic bronchitis Thorax 2003 58 680 685 12885984 10.1136/thorax.58.8.680 PMC1746781

[B45] Stone H McNab G Wood AM Stockley RA Sapey E Variability of sputum inflammatory mediators in COPD and alpha1-antitrypsin deficiency Eur Respir J 2012 40 561 569 22700846 10.1183/09031936.00162811

[B46] Jadad AR Moore RA Carroll D Jenkinson C Reynolds DJ Gavaghan DJ McQuay HJ Assessing the quality of reports of randomized clinical trials: is blinding necessary? Control Clin Trials 1996 17 1 12 8721797 10.1016/0197-2456(95)00134-4

[B47] Hozo SP Djulbegovic B Hozo I Estimating the mean and variance from the median, range, and the size of a sample BMC Med Res Methodol 2005 5 13 15840177 10.1186/1471-2288-5-13 PMC1097734

[B48] Higgins JP Thompson SG Deeks JJ Altman DG Measuring inconsistency in meta-analyses BMJ 2003 327 557 560 12958120 10.1136/bmj.327.7414.557 PMC192859

[B49] Begg C Mazumdar M Operating characteristics of a rank correlation test for publication bias Biometrics 1994 50 1088 1101 7786990

[B50] Egger M Smith G Schneider M Minder C Bias in meta-analysis detected by a simple, graphical test Br Med J 1997 315 629 634 9310563 10.1136/bmj.315.7109.629 PMC2127453

[B51] Metso T Rytil P Peterson C Haahtela T Granulocyte markers in induced sputum in patients with respiratory disorders and healthy persons obtained by two sputum-processing methods Resp Med 2001 95 48 55 10.1053/rmed.2000.0970 11207017

[B52] Miller M Cho JY Pham A Friedman PJ Ramsdell J Broide DH Persistent airway inflammation and emphysema progression on CT scan in ex-smokers observed for 4 years Chest 2011 139 1380 1387 20966041 10.1378/chest.10-0705 PMC3109645

[B53] Hill AT Campbell EJ Bayley DL Hill SL Stockley RA Evidence for excessive bronchial inflammation during an acute exacerbation of chronic obstructive pulmonary disease in patients with alpha (1)-antitrypsin deficiency (PiZ) Am J Respir Crit Care Med 1999 160 1968 1975 10588615 10.1164/ajrccm.160.6.9904097

[B54] Hurst JR Perera WR Wilkinson TM Donaldson GC Wedzicha JA Systemic and upper and lower airway inflammation at exacerbation of chronic obstructive pulmonary disease Am J Respir Crit Care Med 2006 173 71 78 16179639 10.1164/rccm.200505-704OC

[B55] Tsoumakidou M Tzanakis N Chrysofakis G Siafakas NM Nitrosative stress, heme oxygenase-1 expression and airway inflammation during severe exacerbations of COPD Chest 2005 127 1911 1918 15947302 10.1378/chest.127.6.1911

[B56] Bathoorn E Liesker JJW Postma DS Koeter GH van der Toorn M van der Heide S Ross HA van Oosterhout AJM Kerstjens HAM Change in inflammation in out-patient COPD patients from stable phase to a subsequent exacerbation Int J COPD 2009 4 101 109 10.2147/copd.s4854 PMC2672798 19436694

[B57] Gompertz S Stockley RA A randomized, placebo-controlled trial of a leukotriene synthesis inhibitor in patients with COPD Chest 2002 122 289 294 12114372 10.1378/chest.122.1.289

[B58] Gronke L Beeh KM Cameron R Kornmann O Beier J Shaw M Holz O Buhl R Magnussen H Jorres RA Effect of the oral leukotriene B4 receptor antagonist LTB019 on inflammatory sputum markers in patients with chronic obstructive pulmonary disease Pulm Pharmacol Ther 2008 21 409 417 18063399 10.1016/j.pupt.2007.10.007

[B59] Seemungal TA Wilkinson TM Hurst JR Perera WR Sapsford RJ Wedzicha JA Long-term erythromycin therapy is associated with decreased chronic obstructive pulmonary disease exacerbations Am J Respir Crit Care Med 2008 178 1139 1147 18723437 10.1164/rccm.200801-145OC

[B60] Bekçi T Kurtipek E Kesli R Maden E Teke T The Effect of Telithromycin on Infammatory Markers in Chronic Obstructive Pulmonary Diseases European J Gen Med 2009 6 218 222

[B61] Hill AT Bayley DL Campbell EJ Hill SL Stockley RA Airways inflammation in chronic bronchitis: the effects of smoking and alpha1-antitrypsin deficiency Eur Respir J 2000 15 886 890 10853853 10.1034/j.1399-3003.2000.15e12.x

[B62] Ronchi MC Piragino C Rosi E Amendola M Duranti R Scano G Role of sputum differential cell count in detecting airway inflammation in patients with chronic bronchial asthma or COPD Thorax 1996 51 1000 1004 8977600 10.1136/thx.51.10.1000 PMC472648

[B63] Klebanoff SJ Hamon CB Role of myeloperoxidase-mediated antimicrobial systems in intact leukocytes J Reticuloendothel Soc 1972 12 170 196 5075520

[B64] Johnson KJ Fantone JC 3rd Kaplan J Ward PA In vivo damage of rat lungs by oxygen metabolites J Clin Invest 1981 67 983 993 6894154 10.1172/JCI110149 PMC370656

[B65] Cantin AM North SL Fells GA Hubbard RC Crystal RG Oxidant-mediated epithelial cell injury in idiopathic pulmonary fibrosis J Clin Invest 1987 79 1665 1673 3034979 10.1172/JCI113005 PMC424497

[B66] Sutherland ER Martin RJ Airway inflammation in chronic obstructive pulmonary disease: comparisons with asthma J Allergy Clin Immunol 2003 112 819 827 quiz 828 14610463 10.1016/S0091

[B67] Fens N de Nijs SB Peters S Dekker T Knobel HH Vink TJ Willard NP Zwinderman AH Krouwels FH Janssen HG Lutter R Sterk PJ Exhaled air molecular profiling in relation to inflammatory subtype and activity in COPD Eur Respir J 2011 38 1301 1309 21700610 10.1183/09031936.00032911

[B68] Yasui K Agematsu K Shinozaki K Hokibara S Nagumo H Nakazawa T Komiyama A Theophylline induces neutrophil apoptosis through adenosine A2A receptor antagonism J Leukoc Biol 2000 67 529 535 10770286 10.1002/jlb.67.4.529

[B69] Yasui K Agematsu K Shinozaki K Hokibara S Nagumo H Yamada S Kobayashi N Komiyama A Effects of theophylline on human eosinophil functions: comparative study with neutrophil functions J Leukoc Biol 2000 68 194 200 10947063

[B70] Iiboshi H Ashitani J Katoh S Sano A Matsumoto N Mukae H Nakazato M Long-term treatment with theophylline reduces neutrophils, interleukin-8 and tumor necrosis factor-alpha in the sputum of patients with chronic obstructive pulmonary disease Pulm Pharmacol Ther 2007 20 46 51 16413994 10.1016/j.pupt.2005.11.008

[B71] Confalonieri M Mainardi E Della Porta R Bernorio S Gandola L Beghe B Spanevello A Inhaled corticosteroids reduce neutrophilic bronchial inflammation in patients with chronic obstructive pulmonary disease Thorax 1998 53 583 585 9797758 10.1136/thx.53.7.583 PMC1745263

[B72] Brightling CE McKenna S Hargadon B Birring S Green R Siva R Berry M Parker D Monteiro W Pavord ID Bradding P Sputum eosinophilia and the short term response to inhaled mometasone in chronic obstructive pulmonary disease Thorax 2005 60 193 198 15741434 10.1136/thx.2004.032516 PMC1747331

[B73] Ford PA Durham AL Russell RE Gordon F Adcock IM Barnes PJ Treatment effects of low-dose theophylline combined with an inhaled corticosteroid in COPD Chest 2010 137 1338 1344 20299628 10.1378/chest.09-2363

[B74] Ford-Hutchinson AW Bray MA Doig MV Shipley ME Smith MJ Leukotriene B, a potent chemokinetic and aggregating substance released from polymorphonuclear leukocytes Nature 1980 286 264 265 6250050 10.1038/286264a0

[B75] Feinmark SJ Lindgren JA Claesson HE Malmsten C Samuelsson B Stimulation of human leukocyte degranulation by leukotriene B4 and its omega-oxidized metabolites FEBS Lett 1981 136 141 144 6274698 10.1016/0014-5793(81)81233-1

[B76] Hubbard RC Fells G Gadek J Pacholok S Humes J Crystal RG Neutrophil accumulation in the lung in alpha 1-antitrypsin deficiency. Spontaneous release of leukotriene B4 by alveolar macrophages J Clin Invest 1991 88 891 897 1653278 10.1172/JCI115391 PMC295476

[B77] Sallenave JM Shulmann J Crossley J Jordana M Gauldie J Regulation of secretory leukocyte proteinase inhibitor (SLPI) and elastase-specific inhibitor (ESI/elafin) in human airway epithelial cells by cytokines and neutrophilic enzymes Am J Respir Cell Mol Biol 1994 11 733 741 7946401 10.1165/ajrcmb.11.6.7946401

