# Supplementary material for: Sputum myeloperoxidase in chronic obstructive pulmonary disease
Source: Eur J Med Res. 2014 Mar 3;19(1):12. doi: 10.1186/2047-783X-19-12 (PMC4016613; doi:10.1186/2047-783X-19-12)
Supplement: Additional file 1 — Table S1. Studies included in the meta-analysis examining myeloperoxidase (MPO) levels in chronic obstructive pulmonary disease (COPD) patients and healthy subjects and asthmatics. Data are expressed as *mean ± SEM; #mean ± SD; &medians; ^ interquartile ranges; $:95% confidence interval. M, male; F, female; n, the number of participants; FEV1, forced expiratory volume in one second; ELISA, enzyme-linked immunosorbent assay; DDT, dithiothreitol. Table S2. Studies included in the meta-analysis examining myeloperoxidase (MPO) levels in stable COPD and during acute exacerbations. Data are expressed as *mean ± SEM; #mean ± SD; &medians; ^ interquartile ranges; $:95% confidence interval. COPD, chronic obstructive pulmonary disease; M, male; F, female; n, the number of participants; FEV1, forced expiratory volume in one second; AAT, α1-antitrypsindeficiency; ELISA, enzyme-linked immunosorbent assay; ICS, inhaled corticosteroids. Table S3. Studies included in the meta-analysis examining myeloperoxidase (MPO) levels in COPD patients before and after medicine treatment. Data are expressed as *mean ± SEM; #mean ± SD; &medians; ^ interquartile ranges; $:95% confidence interval. COPD, chronic obstructive pulmonary disease; M, male; F, female; n, the number of participants; FEV1, forced expiratory volume in one second; ELISA, enzyme-linked immunosorbent assay. [file 2047-783X-19-12-S1.docx]

**Additional material files**

**Additional file 1：**Studies included in the meta-analysis examining MPO levels in COPD patients and healthy subjects and asthmatics

| **Study (year )** | **Group** | **N(M/F)** | **Age(y)** | **%pred FEV1** | **Clinical feature** | **Method of collecting and treating with stupum** | **Smoking states (Current/exsmoking/nosmoking)** | **MPO value** | **MPO measurement** |
| --- | --- | --- | --- | --- | --- | --- | --- | --- | --- |
| **Keatings**  **1997[14]** | COPD | 16(-/-) | 65.1±2.4* | 42.3±4.6* | stable | Inhale 3.5%saline  DTT | >10pack-years | 9.1±3.6 **(mg/L)*** | commercially available radioimmunoassay (RIA) |
|  | Asthma | 25 (-/-)23 | 29.0±1.4* | 97.8±2.6* | Stable |  | Nonsmoking | 1.1±0.3**(mg/L)*** |  |
|  | Healthy | 16 (13) | 37.8±3.7* | 101.6±2.7* | --- |  | Nonsmoking | 0.2±0.1**(mg/L)*** |  |
| **Yamamoto**  **1997[15]** | COPD | 33(31/2) | 68.8± 1.5* | 45.1±2.8* | stable | spontaneously  PBS | 11 /22 /0 | 83.9±4.9 ng/mL* | ELISA (MPO-EIA kit) |
|  | Asthma | 30(18/12) | 48.1±2.4* | 82.5±2.8* |  |  | 5/7/18 | 83.3±8.7 ng/mL* |  |
|  | Healthy | 12(9/3) | 43.1±5.3* | Not done | --- | inhaled 3% hypertonic saline | 5/0/7 | 42.6±5.7 ng/mL* |  |
| **Gompertz**  **2006[16]** | healthy control subjects | 14(4/10) | 45.6(5.2) | 92.5(3.2) | Stable | inhaled hypertonic saline | 0/3/11 | 0.3±0.02*(U/ml) | Chromogenic assay |
|  | AATD subjects with no lung disease | 9(7/2) | 39.8(2.4) | 113.1(11.6) |  |  | 0/5/4 | 0.3±0.05*(U/ml) |  |
|  | COPD without AAT | 22(12/10) | 65.8(1.4) | 38.9(2.4) |  |  | 4/18/0 | 0.2±0.04*(U/ml) |  |
|  | COPD with AAT no chronic bronchitis | 12(7/5) | 56.3(1.9) | 34.5(4.7) |  |  | 1/10/1 | 0.3±0.05*(U/ml) |  |
|  | COPD with AAT | 14 (11/3) | 52.4(2.1) | 33.2(3.9) |  |  | 2/11/1 | 0.6±0.13*(U/ml) |  |
| **Gorska**  **2008[17]** | COPD | 17(10/7) | 56.8 ±11.2# | 73 ±19# | stable | hypertonic NaCl solutions  DTT | 17/0/0 | 60.7 ±70.1 pg/ml# | MPO-EIA kit |
|  | Asthma | 22(12/10) | 36.1 ±14.5# | 84 ±17# |  |  | 10/12/0 | 92.1 ±81.3 pg/ml# |  |
| **Mesto**  **2001[52]** | Healthy | 42(9/33) | 42.0(29-61)& | n.d | -- | inhaled 3% NaCl solution  DTT | 1/0/0 | 178 (<80-1524)ug/l& | a double-antibody RIA kit (MPO RIA) |
|  | COPD | 8(3/5) | 68.2(58-76 | 42.7(27-66)& | Stable |  | 8/0/0 | 7125(395-58000) ug/l& |  |
|  | Asthma | 17(7/10) | 42.2(18-72 | 85(69-101) |  |  | 6/0/0 | 192(<80-21800) ug/l& |  |
| **Miller**  **2011[53]** | COPD-Eemphysema | 10(9/1) | 69±2# | 44±4# | Stable | --  -- |  | 77.64±35 |  |
|  | Control (smoker) | 8(4/4) | 52±3# | 97±5# |  |  | 8 | 11.32±7.38 | ELISA |
|  | Control(nonsmoker) | 7(1/6) | 50±4# | 98±5# |  |  | 0 |  |  |
| **Hill**  **1999[54]** | COPD with AAT deficiency | 11(8/3) | 48.9±2.9* | 36.3±7.4* | Acute Exacerbations | -- | 3/7/1 | 1.4±0.4U/ml* | the substrate O-dianisidine dihydrochloride |
|  | COPD withoutl AAT deficiency | 11(7/4) | 67.6±2.0* | 38.0±5.7* |  |  | 4/7/0 | 1.0±0.3U/ml* |  |
| **Hill**  **2000[62]** | COPD with AAT deficiency | 39(30/9) | 50±1.5* | 27.4±3.0* | stable | -- | 5/34/0 | 0.6(0.3-2.2)^ | the substrate O-dianisidine dihydrochloride |
|  | COPD without AAT deficiency | 42(27/15) | 67.1±1.2* | 30.1±3.1* |  |  | 22/20/0 | 0.2(0.1-0.4)^ |  |

*mean±sem, #mean±SD, &medians , ^ interquartile ranges,$95%conﬁdence interval. MPO: myeloperoxidase, COPD: chronic obstructive pulmonary disease, M: male, F: female, n: the number of participants, FEV1: forced expiratory volume in one second, ELISA: enzyme-linked immunosorbent assay. DDT: dithiothreitol

**Additional file 2：**Studies included in the meta-analysis examining MPO levels in stable COPD and during acute exacerbations

| **Study (year )** | **Group** | **N(M/F)** | **Age(y)** | **%pred FEV1 or FEV1** | **Clinical feature** | **Smoking states (Current/exsmoking/nosmoking)** | **Treatment** | **MPO value**  **(****Acute Exacerbations）** | **MPO value**  **（stable）** | **MPO measurement** |
| --- | --- | --- | --- | --- | --- | --- | --- | --- | --- | --- |
| **Hill**  **1999[54]** | COPD with AATdeficiency | 11(8/3) | 48.9±2.9* | 36.3±7.4* | -- | 3/7/1 | 8 of these ICS  antibiotics | 1.4±0.4  U/ml* | 0.6±0.2  U/ml*(28d) | -- |
| **Aaron**  **2001[19]** | COPD | 50(40/10) | 71.5±10.0 | 0.95±0.31# | stable | All exsmokers | either antibiotics or oral corticosteroids or both, | 4256±2717  ng/ml* | 2063±1300  ng/ml* | a commercially available ELISA |
|  | Of those AE | 14(11/3) | 71.6±7.7 | 0.89±0.24# | AECOPD |  |  |  |  |  |
| **Crooks**  **2000[18]** | COPD | 8(6/2) | 56-73& | 27.9±3.8* | -- | 4/4/0 | Antibiotic  5 ICS | 0.39±0.16  (U/ml)* | 4.20±1.86  (U/ml)* | the substrate o-dianisidine dihydrochloride |
| **Hurst**  **2006[55]** | COPD baseline | 47 | 70.5±7.0 | 37.9±13.6 | -- | -- | -- | 52.1±20.7#  (ng/ml) | 20.8±8.2#  (ng/ml) | commercial sandwich ELISA kits |
|  | exacerbation | 41 | 69.±7.7# | 40±16.4# |  |  |  |  |  |  |
| **Tsoumakidou**  **2005[56]** | COPD | 12(11/1) | 69 ±7# | 40±14% # | --- | --- | systemic steroids SABA/SAMA  oxygen therapy | 39.9 (4.5-64) &  &1μg/g | 11.9 (1-21.7)&  1μg/g | A competitive radioimmunoassay |
| **Bathoorn**  **2009[57]** | COPD | 114(93/21 | 64(59-71) | 63(52-73) | stable | 47/67/0 | --- | 15.1(8.4-56)  μg/ml ^ | 9.2(5.3-31.0)μg/ml^ | ELISA |
|  | of those AE | 45(37/8) | 65(58-71) | 61(48-73)& | AECOPD | 21/24/0 |  |  |  |  |

*mean±sem, #mean±SD, &medians , ^ interquartile ranges, $:95%conﬁdence interval. MPO: myeloperoxidase, COPD: chronic obstructive pulmonary disease, M: male, F: female, n: the number of participants, FEV1: forced expiratory volume in one second, AAT: α1-antitrypsindeficiency, ELISA: enzyme-linked immunosorbent assay. ICS: inhaled corticosteroids.

**Additional file 3：**Studies included in the meta-analysis examining MPO levels in COPD patients before and after steroid treatment

| **Study (year )** | **Group** | **N(M/F)** | **Age(y)** | **%pred FEV1** | **Clinical feature** | **Smoking states (Current/exsmoking/nosmoking)** | **Treatment** | **MPO value**  **(before treatment)** | **MPO value**  **(after treatment)** | **MPO measurement** |
| --- | --- | --- | --- | --- | --- | --- | --- | --- | --- | --- |
| **Culpitt**  **2002[21]** | COPD | 25(-/-) | 62 ±2 | 20-68% | stable | 11/14/0 | Theophylline  (150–300 mg BID) *4wks | 147.94±67.4  (ng/ml) | 98.67±61.8  (ng/ml) | kits (R&D Systems) |
| **Kobayashi**  **2004[20]** | COPD | 6(6/0) | 71.0±2.6* | 63.1±6.5 | stable | 0/6 | Theophylline 400mg/d *4wks | 15.02±5.58*mg/L | 7.38±4.97* | Radio immunoassay(RIA) |
| **Kanehara**  **2008[22]** | COPD | 26(20/6) | 77.3±3.31 | 83.8±16.3# | stable | 4/14/8 | Theophylline 400mg/d*8wks | 64.18±63.2 | 49.47±47.89 | ELISA kits |
| **Barczyk**  **2004[24]** | COPD | 18(16/2) | 67(48-77)& | 53.2(27.8-79.7) | stable | -- | Oral prednisone, 0.5 mg/kg/d for 2 weeks. | 2.54(1.49-12.58)  μg/mL | 1.79(1.32-3.57)&  μg/mL | enzyme-linked immunosorbent assay |
| **Llewellyn-jones 1996[26]** | COPD | 8(4/4) | 65±7 | -- | stable | -- | Inhaled Fluticasone Propionate 1.5mg/d *8wks | 0.09±0.02 (nm)* | 0.072±0.02* | the substrate O-dianisidine dihydrochloride |
| **Boorsma**  **2008[25]** | COPD | 19(13/6) | 63.1±6.2# | 65.1 ± 22.0 | stable | 13/6 | Prednisolone 30mg/d*3wks | 7.2 (1.8–22.6) (µg/g) | 10.2 (4.0–42.8) (µg/g)^ | -- |
|  |  |  |  |  |  |  | Budesonide 400 µg twice daily*3months | 7.0 (1.7–69.0)  (µg/g) | 11.1 (3.5–19.7) (µg/g)^ |  |
| **Keatings**  **1997 [23]** | COPD | 15(9/6)(13) | 35.1±1.3 | 1.02±0.17 | stable | 6/9 | Budesonide 800mg twice daily*2wks | 8.05±1.49  (mg/l)* | 2.97±0.39  (mg/l)* | RIA |
| **Gompertz**  **2002[58]** | COPD | 8(7/1) | 66.5±5.2# | 30.2±12.7# |  | 6/1 | BAYx1005 (500 mg bid) | 0.71(0.2-2.43)  U/ml^ | 0.47(0.22-0.85) U/ml ^ | A chromogenic substrate assay |
|  |  | 9(6/3) | 67.9±7.0# | 40.2±15.6# |  | 4/4 |  |  |  |  |
| **Gronke**  **2008[59]** | COPD | 24(18/6) | 63.9±5.O# | 56.8±9.7# | Stable | -- | LTB019* 4weeks | 10.55(1.1-67.9)&  μg/ml | 7.80 (0.1–68.5)& μg/ml | ELISA |
| **Seemungal**  **2008[60]** | COPD | 53(33/20) | 66.54±8.1# | 49.25±17.3# | stable | - | Erythromycin *12months | 13.3(8.3-18.2) $  (n=39) | 16.1(6.3-25.8) $  n=25 | ELISA |
| **Bekçi**  **2009[61]** | COPD | 17(-/-) | 60.2±10.1# | 50.8±0.55# | stable | -- | Telithromycin*10d | 0.65± 0.55#µM | 0.76±0.63# | ELISA |

*mean±sem, #:mean±SD, &medians , ^ interquartile ranges, $95%conﬁdence interval. MPO: myeloperoxidase, COPD: chronic obstructive pulmonary disease, M: male, F: female, n: the number of participants, FEV1: forced expiratory volume in one second, ELISA: enzyme-linked immunosorbent assay.
